# Repeat what after whom? Exploring variable selectivity in a cross-dialectal shadowing task

**DOI:** 10.3389/fpsyg.2015.00546

**Published:** 2015-05-12

**Authors:** Abby Walker, Kathryn Campbell-Kibler

**Affiliations:** ^1^Department of English, College of Liberal Arts and Human Sciences, Virginia Polytechnic Institute and State UniversityBlacksburg, VA, USA; ^2^Department of Linguistics, The Ohio State UniversityColumbus, OH, USA

**Keywords:** accommodation, single-word shadowing, U.S. English, New Zealand English, AXB task

## Abstract

Twenty women from Christchurch, New Zealand and 16 from Columbus Ohio (dialect region U.S. Midland) participated in a bimodal lexical naming task where they repeated monosyllabic words after four speakers from four regional dialects: New Zealand, Australia, U.S. Inland North and U.S. Midland. The resulting utterances were acoustically analyzed, and presented to listeners on Amazon Mechanical Turk in an AXB task. Convergence is observed, but differs depending on the dialect of the speaker, the dialect of the model, the particular word class being shadowed, and the order in which dialects are presented to participants. We argue that these patterns are generally consistent with findings that convergence is promoted by a large phonetic distance between shadower and model (Babel, 2010, contra Kim et al., 2011), and greater existing variability in a vowel class (Babel, 2012). The results also suggest that more comparisons of accommodation toward different dialects are warranted, and that the investigation of the socio-indexical meaning of specific linguistic forms in context is a promising avenue for understanding variable selectivity in convergence.

## Introduction

A substantial body of work spanning multiple fields and at least four decades has documented the tendency for speakers to adjust their speech in relation to their interlocutors, most often by becoming more like them. These effects have been shown to emerge in spontaneous, interactive speech (Natale, 1975; Gregory and Webster, 1996; Willemyns et al., 1997; Pardo, 2006), in speech tasks with elements of interaction (Giles et al., 1973; Natale, 1975) and in socially impoverished lab-based shadowing tasks (Goldinger, 1998; Shockley et al., 2004; Babel, 2010). Research has shown lingering effects of accommodation beyond immediate exposure (Delvaux and Soquet, 2007), and a number of researchers have argued that accommodative processes play a critical role in sound change (Pardo, 2006; Delvaux and Soquet, 2007; Trudgill, 2008; Smith, 2013).

Two distinct effects seem to be involved in accommodative processes. First, individuals often attempt to appeal socially to interlocutors by emphasizing similarities (Giles et al., 1973; Giles and Powesland, 1975; Bell, 1984), although their ability to diverge when appropriate (Bourhis et al., 1979) suggests that this forms part of a larger system of stylistic self-presentation (Coupland, 2007). Second, linguistic production systems appear to be impacted directly by the perceptual process, causing productions to slightly increase resemblance of recently heard tokens, so that convergence is observed even absent clear interactional motivation (Goldinger, 1998; Pickering and Garrod, 2004). Even in contexts lacking interactional motivation, however, accommodation has been shown to be subject to social attitudes (Babel, 2010, 2012; Abrego-Collier et al., 2011). In a particularly thorough exploration, Yu et al. (2013) document the importance of both situationally-based social attitudes and individual differences in personality and cognition, and the lack of effects from the broad demographic categories of gender and sexual orientation. Babel (2012) has argued that although speech accommodation is the product of a primary, automatic alignment mechanism (Pickering and Garrod, 2004, 2006; Gentilucci and Bernardis, 2007), much like non-speech accommodation (Dijksterhuis and Bargh, 2001), social factors may act to inhibit this otherwise automatic behavior^1^.

In addition to selectivity in regards to who they accommodate toward, speakers may show selectivity in the features of the speech signal that they accommodate on (Babel, 2012). Acoustic analyses of shadowed speech show that speakers converge on another speaker's f0 (Goldinger, 1997), intensity (Natale, 1975; Gregory and Hoyt, 1982), vowel duration (Hargreaves, 1960; Webb, 1970), formants (Tilsen, 2009; Babel, 2012), VOT (Shockley et al., 2004; Nielsen, 2007, 2008, 2011), pre-aspiration timing (van Dommelen et al., 2011), and long term average spectra (Gregory et al., 1993; Gregory and Webster, 1996). However, studies comparing accommodation *across* different variables show variability in terms of whether shifting is observed, the direction of shift and the degree of shift (Putman and Street, 1984; Goldinger, 1998; Babel, 2010, 2012; Lewandowski and Dogil, 2010; Pardo, 2010; Pardo et al., 2010, 2012, 2013; Lelong and Bailly, 2011; Levitan and Hirschberg, 2011). Furthermore, multiple studies have documented influence of the specific vowel class on how much convergence is observed, though which vowels facilitate convergence varies across studies (Babel, 2010, 2012; Pardo, 2010).

At least some of this variable-selectivity appears to be due to constraints coming from the shadower's linguistic system. For example, looking at shifting within American English, Babel (2009) found that Californian speakers most shadowed the low vowels of two Californian male speakers, compared to their high or mid vowels. She argues that this may be because lower vowels have inherently larger production spaces, licensing participants to make large shifts on these vowels while still staying in their personal phonetic repertoire. Kim et al. (2011) make a similar argument for why they found speakers converging more to interlocutors who shared the same dialect background as them, compared to interlocutors who had a different dialect history.

A tendency to stay within one's own repertoire initially seems contrary to another effect researchers have noted: speakers shift more if the model is further away (Trudgill, 1981; Babel, 2010, 2012). However, these two factors could work together as an interacting constraint: shadowers are more likely to shift toward big differences, if their pre-existing repertoires allow it. A reason that bigger differences may lead to bigger shifts could be because participants have to be able to notice differences in order to shift. Namy et al. (2002) argue that the reason they observe more accommodation by women may be because women are more perceptive of accommodation. Babel et al. (2013) directly explore the relationship between an individual's response in a listening and production task, and find weak evidence that participants who adapt more perceptually also show greater shifts in production.

Applying this to individual variables, we might expect speakers to shift on the variables they are best able to hear. Certainly, it appears to be the case the cultural awareness of a variant, usually measured in terms of how likely speakers are to remark on it, impacts how much they will shift toward it. But whether such awareness inhibits or facilitates accommodation is unclear. For example, Babel (2010) found that New Zealand participants converged toward an Australian speaker's dress^2^ vowel in a shadowing task, but not to kit and trap. She argues that this is because the dress difference is large in the two varieties, but—unlike the also very different kit vowel—is a difference below the level of consciousness. This mirrors arguments made in the second dialect acquisition literature (Sankoff, 2004; Nycz, 2013). However, it is worth noting that in related work on dialect priming, Drager et al. (2010) found that priming Australia caused New Zealand speakers to shift their kit vowel toward Australian English, but not trap or dress. They argue that the social saliency of the kit vowel—the shibboleth marker of NZ and Australian dialects—is why it was the only vowel that shifted toward a more Australian realization (cf. Trudgill, 1981). It remains to be seen how these apparently conflicting results are to be reconciled, but one promising avenue lies in the differences between the tasks, and particularly in whether the linguistic shift is prompted by conceptual primes or actual linguistic tokens.

Differential shifting may also be observed depending on the phonemic status of the shift. While Mitterer and Ernestus (2008) only find effects of phoneme level shifting, subphonemic shifting has been observed in a number of other studies (i.e., Shockley et al., 2004; Nielsen, 2008, 2011). However, Kim and de Jong (2007) argue that a speaker's own phonological inventory will affect whether they make gradient or categorical shifts. Nielsen (2011) showed that American English speakers adapted to lengthened but not shortened VOT in voiceless stops, and while she argues that the mechanism behind the difference is unclear, the fact that a shortened but not lengthened VOT encroaches on a phonemic boundary suggests that “phonetic imitation is a process which is sensitive to phonological structure” (p. 137).

One final factor that is not usually considered in the accommodation literature is the social meaning of a given variant. An extensive body of work on sociolinguistic variation, particularly that identified as “third wave” (Eckert, 2005), has shown that speakers attach complex locally defined meanings to linguistic cues, treating specific variants as loci for meaning rather than only evaluating whole varieties (see, e.g., Eckert, 2000; Zhang, 2005; Mendoza-Denton, 2008; Campbell-Kibler, 2009). Recent perceptual work in sociolinguistics has shown how manipulating a single variable can result in changes to perceived social attributes such as age, ethnicity, social class, and intelligence (e.g., Fridland et al., 2004; Walker, 2007; Szakay, 2008). Given this understanding, it is likely that the effects of recent exposure must interact with the larger context of the linguistic production of self, a context which is likely to impose constraints on the production of specific indexically loaded forms. The fact that very recent work has also shown that the particular way a variant affects how a speaker is perceived can depend on both the speaker and the listener (Campbell-Kibler, 2007; Pharao et al., 2014; Walker et al., 2014) suggests that the social loading of a variable could additionally be mediated by its specific context.

Since we observe selectivity depending on the speaker, and selectivity depending on the variable, it is interesting to consider the ways in which the two might interact. That is, does the variable in combination with the model speaker matter in whether we observe phonetic convergence? Comparing accommodation across dialects is an excellent place to explore such an interaction because it often consists of both social and linguistic considerations: the social associations of a dialect, the social saliency of the variables, the restricted phonetic space of a shadower's dialect, and/or intervening phonological inventories and boundaries. While accommodation work coming from the Communicative Accommodation Theory tradition has largely focused on speech and interaction across ethnolinguistic boundaries (e.g., Giles et al., 1973; Doise et al., 1976), most accommodation work in phonetics/pyscholinguistics has examined accommodation to speech from the same dialect or variety as the shadowers (e.g., Goldinger, 1998) or which differs in a single, controlled acoustic feature (e.g., Abrego-Collier et al., 2011). Only a small body of work has examined lab-based convergence across dialect boundaries, but it has supported the more general observations of weak but significant convergence. In addition to Babel's work on New Zealand English, Delvaux and Soquet (2007) have found cross-dialect accommodation in regional varieties of Belgian French, while Phillips and Clopper (2012) found no acoustic evidence of accommodation (though weak perceptual evidence). Kim et al. (2011), comparing accommodation between D(ialect)1-D1 speakers, D1-D2 speakers, and L(anguage)1-L2 speakers find convergence in the first, but not the two latter pairings, summarizing that their results “generally support the hypothesis that closer interlocutor language distance facilitates phonetic convergence between talkers in conversations” (p. 141).

In the present study, we investigate the relationship between social and variable selectivity by examining cross-dialectal accommodation in a shadowing task. Specifically, we asked U.S. Midland and New Zealand participants to shadow four model speakers, from the U.S. Midland, the U.S. Inland North, Australia, and New Zealand. We contrast geographically local but linguistically distinct boundaries (northern vs. central/southern Ohio; New Zealand vs. Australia) with linguistically and geographically large boundaries [American vs. Australian and New Zealand (Antipodean)]. At a general level, based on Kim et al.'s (2011) findings, we would expect to see the most shifting to speakers' own dialects and no convergence to the most distant dialects. But if phonetic distance does not inhibit but instead facilitates shifting (Babel, 2012), we would expect the reverse to be true. A more nuanced approach may be possible, however, by probing accommodative behavior on specific variables in order to tease apart the role of talker-shadower phonetic distance and other factors such as the variability of a given variable in the shadower's variety.

We supplement our acoustic analysis with a perceptual analysis of accommodation, using the AXB task (Goldinger, 1998; Pardo, 2006; Babel, 2012). Convergence is more often attested in studies where it is evaluated using perceptual instead of acoustic measures (Phillips and Clopper, 2012; Pardo, 2013). An AXB task will allow us to assess whether accommodation on any acoustic dimension is found, but cannot tell us specifically which features are shifted. However, we can investigate possible acoustic features motivating listener judgments by including vowel formant values as predictors in a model of AXB responses. We expect that these features are likely to correlate with AXB judgments, but also that convergence or divergence will be observed on other acoustic dimensions that listeners are sensitive to, which would be especially interesting should it interact with shadower dialect, shadowee dialect, or vowel class.

## Experiment one: acoustic analysis of a shadowing task

### Materials

Ten (C)CVC(C) words were selected for each class dress, kit, trap, bath^3^, lot, price, and near (see Appendix A). Four college-educated, white females aged between 20 and 30 years were recorded reading the stimuli. One speaker came from Perth, Australia (Western Australian Dialect), one from Christchurch, New Zealand (New Zealand English Dialect), one from the suburbs of Cincinnati, Ohio (U.S. Midlands Dialect), and one from Akron, Ohio (U.S. Inland North Dialect). The Antipodean speakers were recorded in a quiet room at the University of Canterbury (NZ) using a head-mounted microphone. Recordings were made directly onto a Toshiba laptop with Sonic Foundry SoundForge 6.0, linked to the microphone through a USB Pre 1.5 interface (44K, 16bit). The American speakers were recorded in a quiet room at The Ohio State University, using a head mounted microphone attached to an H4 Zoom recorder (44K, 16 bit). Model speakers were intensity leveled prior to presentation.

**Figure 1:**
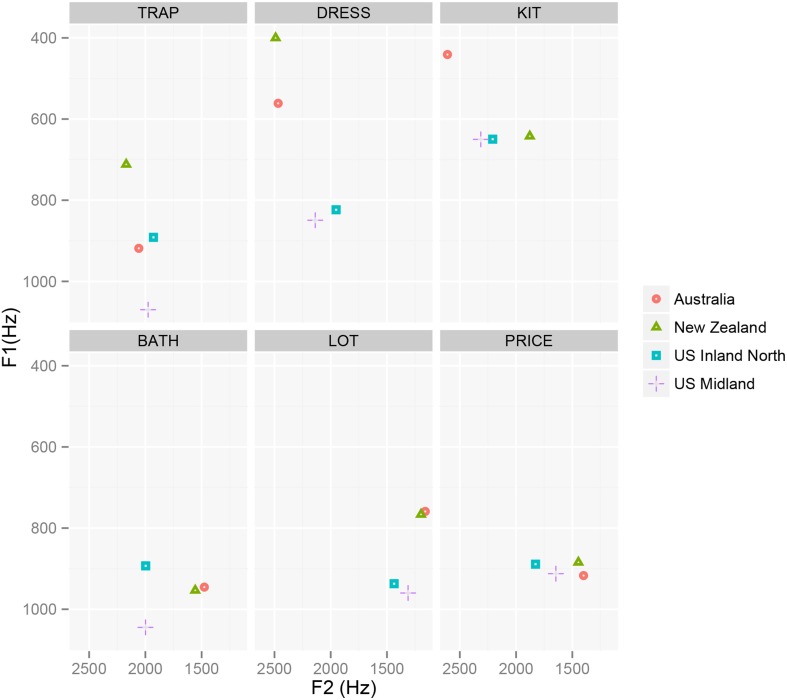
**Mean F1 and F2 for each model speaker by vowel class**. Measurements taken at vowel midpoint, except for the diphthong price, where the measurement was taken 20% into the vowel.

The vowel plots of these speakers are shown in Figure 1 and are fairly representative of their dialect regions. The Antipodeans have almost identical back vowel systems, both having a much higher and backer lot and a backer price nucleus than the Americans, and both having the bath-trap split (Bauer and Warren, 2004; Bradley, 2004), such that their bath approximates the Americans' lot in the vowel space). The Antipodeans differ primarily and substantially in the front vowel system: New Zealanders have considerably raised dress and trap, and a centralized kit (Watson et al., 1998; Cox and Palethorpe, 2008). However, compared to the U.S. Midland speaker, the Australian's kit, dress and trap are all raised.

The biggest difference between the U.S. Midland and U.S. Inland North speakers is in their trap, which is raised in the U.S. Inland North speakers, and typical of the Northern Cities Vowel Shift (Labov et al., 2006). The U.S. Midland lot is also backed compared to the U.S. Inland North (Durian, 2012), and the nucleus of their price is also backer, and closer to the Antipodeans.

**Figure 2:**
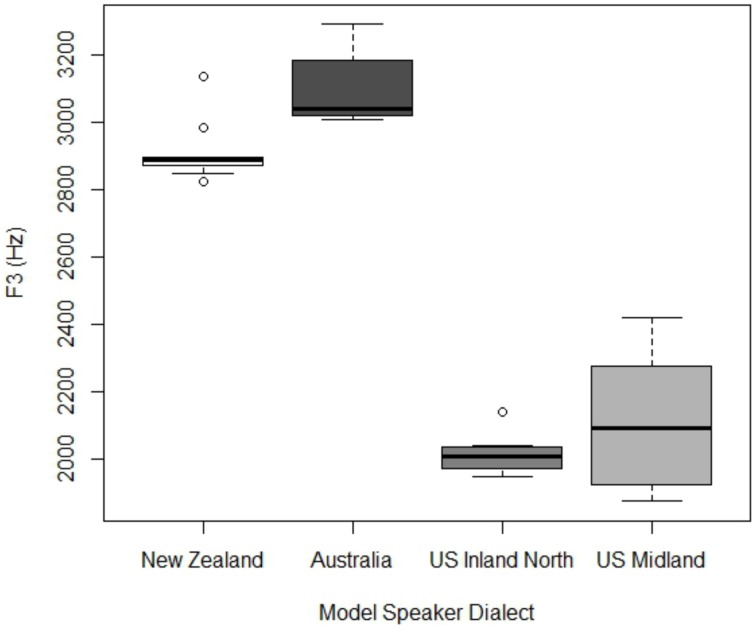
**F3 values for model speaker for NEAR class words, taken at the 65% point in the rhyme**.

Figure 2 shows the F3 values at 65% of the way through the rhyme of near class words. Unsurprisingly, the rhotic American models have lower F3 values than the non-rhotic Antipodeans (New Zealander mean = 2913 Hz; Australian mean = 3108 Hz, U.S. Inland North mean = 2013 Hz, U.S. Midland mean = 2108 Hz). Additionally, the U.S. Inland North speaker has lower F3 than the U.S. Midland speaker.

If accommodation is primarily a function of phonetic distance, where greater distance facilitates more shifting, we would expect to see accommodation by New Zealanders to the Australian model on the front vowels only, and to both American models on all vowels and in rhoticity, but more to the U.S. Midland model than the U.S. Inland model on trap and more overall on dress, which is the most distant vowel between the U.S. and NZ models. We would expect to see converse behavior from the American shadowers to the New Zealanders and the Australians, though less strongly to the Australian model on the front vowels. The American participants, from a U.S. Midland dialect, would only distinguish between the two American dialects on trap/bath, and rhoticity. If it is phonetic *closeness* that facilitates shifting, we could expect to see these patterns reversed. And if the results do not resemble either pattern, it suggests that other/additional factors are influencing convergence.

### Participants

Because gender has been shown to affect convergence (Namy et al., 2002; Pardo, 2006), but was not the object of study here, we limited our data collection to female participants. Twenty female New Zealanders were recruited and run at the University of Canterbury, in Christchurch, New Zealand, and received NZD$10 for their participation. Sixteen female speakers from the Midland dialect region in Ohio were recruited through the Linguistics subject pool at the Ohio State University, and run at OSU, Columbus Ohio.

To quantify the differences between our model speakers and participants, Table 1 shows the mean Euclidean distance in F1-F2 between the participants' base productions and the model productions across the five monophthongs (taken at the vowel midpoint) and price (taken at the vowel 20% point), and the F3 difference for near. The single biggest difference is between the Australian model and the New Zealand participants on kit, but the American participants also show a large difference to the Australian, and the New Zealanders to the Midland model. For dress, there is a symmetrically large difference between the American participants and the Antipodean models, and the New Zealand participants and the American models. For bath, New Zealanders show a large difference to American bath. Finally, the distance between American participants and New Zealand trap is also notable. In terms of rhoticity, the American participants are largely different to the Antipodean models, and the New Zealanders to the Inland North model, but not nearly as much to the Midland model.

**Table 1:** **Mean distance between a participant's and the model's productions, by participant dialect, model dialect, and vowel class**.

**Model Dialect >**	**Australia**		**New Zealand**		**U.S. Inland**		**U.S. Midland**	
**Participant Dialect >**	**Midland**	**NZ**	**Midland**	**NZ**	**Midland**	**NZ**	**Midland**	**NZ**
**F2-F1 SPACE (Hz)**								
bath	354	207	256	164	269	512	260	538
kit	549	760	259	159	178	377	255	469
dress	575	246	677	187	149	612	248	537
trap	302	294	461	184	214	277	255	404
lot	276	176	246	162	161	373	157	311
price	382	238	292	178	234	454	205	347
**F3 DIFFERENCE (Hz)**								
near	852	233	666	48	145	764	218	397

### Procedure

Participants were told they were participating in a “Dialect Identification Task.” After reading the list of target words (and an additional set of point vowels) to get a baseline recording, participants began the shadowing task. The shadowing task consisted of four blocks, one for each speaker. Speech was presented over headphones and the target word also appeared on screen, to avoid ambiguity about the intended word given the considerable difference in vowel systems. Each word appeared on screen at the same time that speakers heard it, and they were asked to repeat the word in their own voice. Participants were told that the goal of the repetition was to allow them to reflect on the differences between their own speech and that of the speaker they heard, and were specifically instructed therefore to not attempt to sound like the speaker they heard. At the end of each block, participants were asked where they thought the speaker was from, what cues they used to judge, and if there was anything else they wanted to say about the speaker. They would then move on to the next speaker.

The presentation of the study as a dialect identification task was primarily so that we could control, across speakers, their understanding of and attention to the different dialects that they heard in the experiment, and the purpose of the task itself. Additionally, it meant that we could investigate any differences based on *perceived* dialect region, and that we received explicit commentary from participants about dialectal features they noticed as being marked. This methodological decision means that participants were possibly more sensitive to comparisons between their own dialect and the model dialect than they would be had we presented the dialects to them unexplained^4^, though it is naive to think that students do not notice (and try to understand why they are being played) different dialects of their own accord. Shadowers were instructed specifically not to imitate in order to limit effects as much as possible to unconscious accommodation. While it is assumed in accommodation (vs. imitation) studies that participants are not consciously imitating models, this instruction is rarely explicitly given to participants. In fact, usually the lack of any instruction is assumed to result in non-explicit imitation (i.e., Namy et al., 2002, p. 425). We included it in our study to not need this assumption^5^, and to provide a more consistent basis for understanding the role of explicit strategy in our results. Our shadowers are likely to have exerted conscious effort to avoid accommodation, an effect likely to fall more heavily on variables they are consciously aware of. While this is important to keep in mind when reflecting on our results, we note that it offers an advantage over studies without such an instruction, where the role of conscious effort is left to the individual subject.

Although no associations were found between the responses in the dialect identification task and the shadowing task, the dialect identifications themselves are worth brief comment. Firstly, all New Zealand participants easily identified both American speakers as being North American, though were unsure where in the U.S. they came from and were mostly unaware that they came from different dialect regions (one participant thought it was the same speaker). Midland speakers showed some more fine-grained categorization, with 44% aware that the U.S. Inland speaker was from north Ohio, and 69% identifying the Midland speaker as from Columbus. The New Zealand speaker was correctly categorized as a New Zealander by all New Zealand participants, though only 60% guessed that the Australian speaker was from Australia (25% said New Zealand and 15% said the UK). Midland responses to both Antipodean models were split between Australia and the UK, with only one person correctly identifying the New Zealand model as a New Zealander.

In terms of the noticeable features of the dialects, most participants in both locations commented on the bath-trap split after shadowing the geographically distant dialects, followed by comments about rhoticity (by New Zealanders more than the Midlanders). The majority of Midlanders commented on the New Zealand model's dress vowel, but only two New Zealanders mentioned the U.S. dress vowels. Midlanders were also sensitive to the raised trap of the U.S. Inland North speaker, while New Zealanders commented frequently on the raised kit vowel of the Australian.

Participants in New Zealand were recorded directly onto a Toshiba laptop with Sonic Foundry SoundForge 6.0, linked to the microphone through a U.S.B Pre 1.5 interface (44 K, 16bit), and participants in Columbus were recorded using a H4 Zoom recorder (44 K, 16 bit). Although the baseline recording was always presented as the first block, the shadowed voices were presented in one of two orders: either the New Zealander, then the Australian, the U.S. Midland, and the U.S. Inland North speaker, or the reverse. In this way, some participants started with the dialect closest to their own, and got progressively further away, while other participants started further away and got progressively closer.

### Data analysis

Sound files were segmented using the Penn Forced aligner (Yuan and Liberman, 2008), then hand corrected. F1, F2, and F3 were extracted (via LPC analysis set to 5 formants under 5500 Hz) and hand-corrected in the Emu Speech Database System (Institute of Phonetics and Speech Processing, LMU Munich 2010). For monophthongs, the vowel midpoint was extracted as the focus for comparison. For the diphthong price, we compared the nuclei by taking the F1 and F2 at the 20% point of the vowel. For comparing levels of rhoticity on near words, we took the F3 value at the 65% point of the rhyme.

For every intact shadowed token whose corresponding baseline utterance was also intact (9956 tokens, 98.8%), we calculated the F1-F2 Euclidean distance for vowels and the F3 distance for near, between model and the shadower's base production, and between the model and the shadower's shadowed production. To measure the change in these distances across the study, we subtracted the Euclidean distance in the baseline task from the distance during the shadowing task. A value of zero means that the distance between the participant and the speaker they were shadowing did not change. A positive value means that the participant became more similar to the shadowee in the shadowing task, which we would interpret as convergence. A negative value means that the participant became more different to the shadowee in the shadowing task, which we would interpret as divergence.

A mixed effects linear regression model was fit to the difference in distance measures for vowel midpoints for all word classes except price and near (where we were interested in diphthong nucleus and F3 values respectively), testing the four-way interaction of regional origin of shadower, condition, order of presentation and word class. Random effects for word and shadower were included, along with a random slope of condition for each. The four way interaction was found to significantly improve the model over the four possible three-way interactions based on pairwise model comparison using R's ANOVA function (*p* < 0.001). Due to the difficulty of interpreting such a complex interaction, this result was taken to motivate separate analyses for the New Zealand and U.S. Midland participants. Each initial model for the two dialect groups included the random effects Subject and Word, each with a random slope for shadowing condition. Fixed effects tested, coded for sum contrasts, were the word class (base group kit^6^), shadowing condition (i.e., was the model the Australian, New Zealander, U.S. Midlander or U.S. Inland Northerner [base group New Zealand for NZ participants, and Midland for Midland participants)], shadowing block order (base group U.S. Midland first for Midland participants, and NZ first for NZ participants), and a three-way interaction between them. We additionally tested the effect of the CELEX log wordform frequency (Baayen et al., 1995). Items were included based on model comparison using R's ANOVA function, retaining those which significantly (α = 0.05) improved the model as a whole.

### Results

#### New Zealand speakers

**Figure 3:**
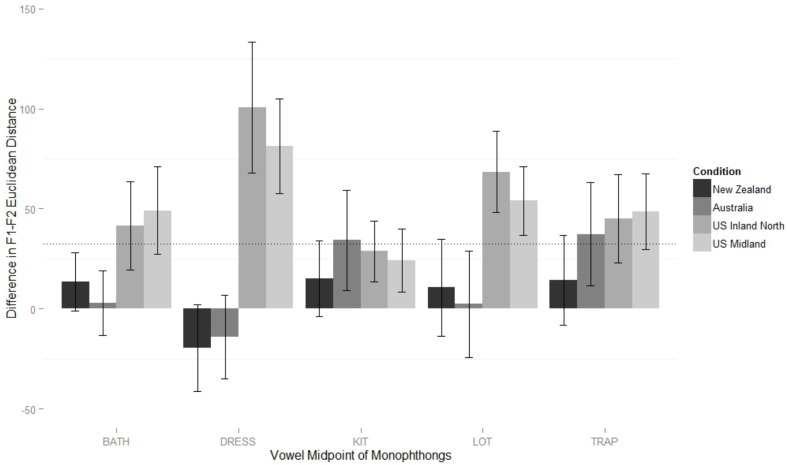
**New Zealander participants' difference in Euclidean distance from models on monophthongs, from baseline reading to shadowed reading**. Values at zero represent no shift, positive values a shift toward the model, and negative values a shift away from the model, compared to the baseline. Measurements are at the vowel 50% point. Error bars mark 95% confidence intervals. Dotted line represents grand mean of the linear regression model.

The final model (Table 2) for the monophthongs of the New Zealand shadowers supported two two-way interactions: between vowel class and condition, and between vowel class and order. Figure 3 shows the first interaction, plotting the Euclidean difference in distance between the speaker and model for the five monophthongs.

**Table 2:** **Summary of best mixed effects models for New Zealand Participants**.

	**Estimate**	**Std. error**	***t*-value**	***p*-value**
**NZ MONOPHTHONG MODEL (DIFFERENCE IN EUCLIDEAN DISTANCE AT VOWEL MIDPOINT)**				
(Intercept)	32.391	6.040	5.362	<0.001^*^
Order = US first	4.609	4.190	1.100	0.271
Word Class = bath	−5.214	6.640	−0.785	0.432
Word Class = dress	3.950	6.362	0.621	0.535
Word Class = lot	1.750	6.344	0.276	0.783
Word Class = trap	6.240	6.344	0.984	0.325
Condition = Australian	−19.283	3.802	−5.072	<0.001^*^
Condition = U.S. Midland	19.577	4.313	4.539	<0.001^*^
Condition = U.S. Inland North	24.770	5.916	4.187	<0.001^*^
Order = US first: Word class = bath	−0.090	4.000	−0.022	0.982
Order = US first: Word class = dress	−5.219	3.822	−1.366	0.172
Order = US first: Word class = lot	−5.246	3.793	−1.383	0.167
Order = US first: Word class = trap	17.472	3.793	4.606	<0.001^*^
Condition = Australia: Word class = bath	−4.519	6.963	−0.649	0.516
Condition = Australia: Word class = dress	−31.062	6.625	−4.689	<0.001^*^
Condition = Australia: Word class = lot	−11.800	6.603	−1.787	0.074
Condition = Australia: Word class = trap	20.082	6.585	3.050	0.002^*^
Condition = Midland: Word class = bath	3.486	6.938	0.502	0.615
Condition = Midland: Word class = dress	23.890	6.645	3.593	<0.001^*^
Condition = Midland: Word class = lot	0.582	6.618	0.088	0.930
Condition = Midland: Word class = trap	−7.509	6.608	−1.136	0.256
Condition = Inland North: Word class = bath	−10.424	6.923	−1.506	0.132
Condition = Inland North: Word class = dress	38.670	6.623	5.839	<0.001^*^
Condition = Inland North: Word class = lot	9.049	6.567	1.378	0.168
Condition = Inland North: Word class = trap	−16.280	6.565	−2.480	0.013^*^
**NZ PRICE MODEL (DIFFERENCE IN EUCLIDEAN DISTANCE AT VOWEL 20%)**				
(Intercept)	9.381	15.896	0.590	0.555
Order = NZfirst	−9.737	12.816	−0.760	0.447
**NZ NEAR MODEL (DIFFERENCE IN F3 DISTANCE AT RHYME 65%)**				
(Intercept)	27.173	20.079	1.353	0.176
Condition = Australia	−9.811	27.193	−0.361	0.718
Condition = U.S. Midland	25.312	26.264	0.964	0.335
Condition = U.S. Inland North	15.808	25.149	0.629	0.530
Order = Midland first	9.695	20.079	0.483	0.629
Condition = Australia: Order = Midland first	−56.150	27.193	−2.065	0.039^*^
Condition = U.S. Midland: Order = Midland first	−5.029	26.264	−0.192	0.848
Condition = U.S. Inland North: Order = Midland first	64.144	23.156	2.770	0.006^*^

*Dependent variable is the difference in Euclidean distance between model and shadower across baseline and shadowing recordings. Random effects = (1 + Condition |Word) + (1 + Condition |Subject)*.

*Asterisks indicate significance at a 0.05 alpha*.

**Figure 4:**
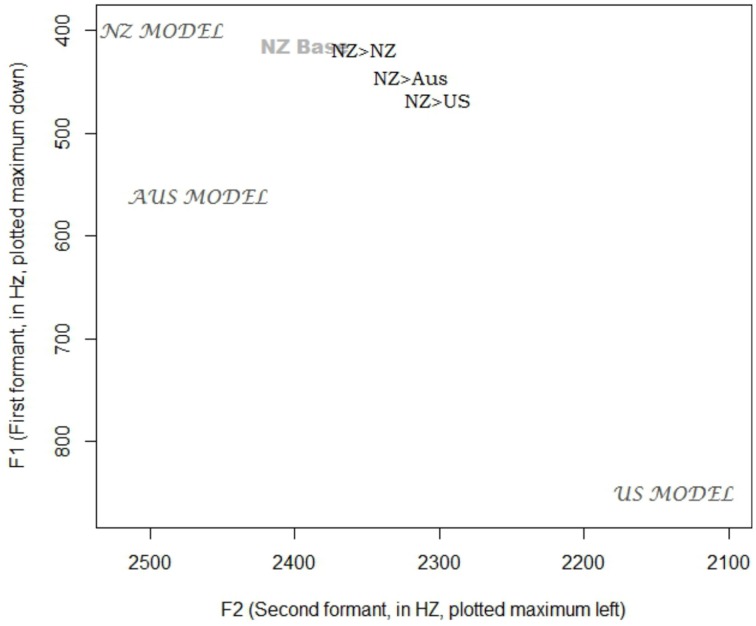
**Mean values for New Zealand participants in their baseline production of DRESS, and their production of DRESS in response to the New Zealand, Australian, and U.S. Midland models (also shown)**.

The grand mean of the model (32 Hz) is significantly larger than zero (*p* < 0.001), showing that overall, New Zealand participants converged during the shadowing task. Relative to the mean, New Zealanders converged significantly more to both American models (Midland β = 20 Hz, *p* < 0.001; North β = 25, *p* < 0.001) and significantly less to the Australian model (β = −19Hz, *p* < 0.001). Two vowel classes mitigate this general effect. For dress, the condition difference is intensified, with extra convergence to the American models (Midland β = 24 Hz, *p* < 0.001; North β = 39 Hz, *p* < 0.001) and significantly less to the Australian model (β = −31 Hz, *p* < 0.001). This exceptional lack of shifting to the Australian (and New Zealand) models, clearly visible in Figure 3, is examined in more detail in Figure 4, which shows the baseline NZ participant productions and their shifts relative to the condition models. The New Zealand model's dress is more innovative than the baseline mean, and the Australian model's dress, while certainly lower than the NZ baseline, is also fronter. The participants response to all input seems to be to lower and back their dress, which results in divergent behavior to the Antipodean models.

The convergence to the Australian model on trap is significantly greater than the mean convergence toward her monophthongs overall (β = 20 Hz, *p* = 0.002), while convergence to the Inland North speaker on trap is significantly *less* than the mean convergence toward her monophthongs overall (β = −16 Hz, *p* = 0.013). The interaction in the model between word class and order is driven by trap, which New Zealand participants converge more toward in general if they hear the Americans first (β = 17 Hz, *p* < 0.001).

**Figure 5:**
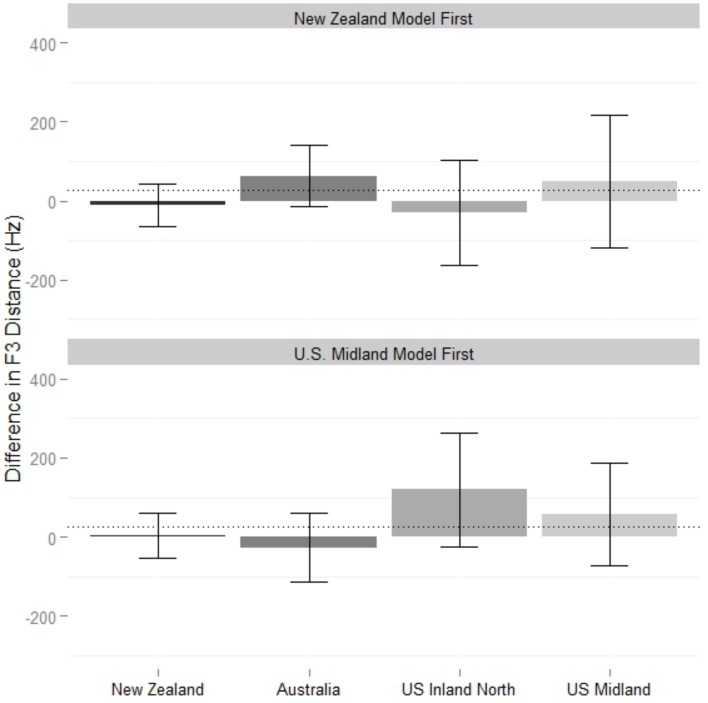
**Difference in F3 distance between New Zealand shadowers and their model across baseline and shadowed tokens on NEAR words. Top panel**: When participants began with the New Zealand block. **Bottom panel**: When participants begin with U.S. Midland block. Error bars represent 95% confidence. Dotted line represents grand mean of model.

Figure 5 shows the difference in F3 distance on near words across conditions. The grand mean of the final model (Table 2) is not significantly different than zero, so in general, New Zealanders did not show convergence to the models' F3 values. However, it includes a significant interaction between block order and condition: participants converge significantly more to the US Inland North model (β = 64 Hz, *p* = 0.006) and converge significantly less to the Australian model (β = −56 Hz, *p* = 0.039) when they hear the American models first. The best price model (Table 2) does not include a condition effect, and in fact there is no evidence that accommodation happened at all (the grand mean is not significantly different than zero).

#### U.S. midland speakers

**Figure 6:**
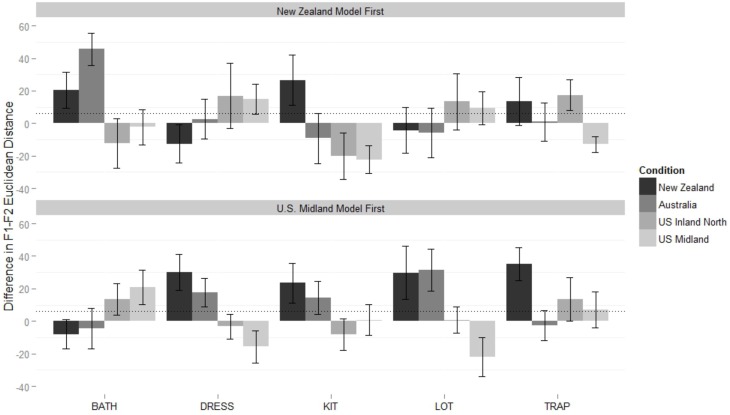
**Midland participants' difference in Euclidean distance from models on monophthongs, from baseline reading to shadowed reading**. Values at zero represent no shift, positive values a shift toward the model, and negative values a shift away from the model, compared to the baseline. **Top panel**: When participants begin with the New Zealand block. **Bottom panel**: When participants begin with Midland block. Error bars mark 95% confidence intervals. Dotted line represents grand means of models.

Unlike the New Zealand model, the grand mean (6 Hz) of the best fit model of the U.S. Midland shadowers (Table 3) is not significantly different than zero (*p* = 0.148), suggesting an overall pattern of non-convergence (Figure 6). This model does, however, include a significant three-way interaction between word class, condition and order. In the bottom panel of Figure 6, we see the shift in monophthongs' midpoint by U.S. Midland speakers when they begin the task shadowing a U.S. Midland speaker (then U.S. Inland North, then Australian, then NZ), and in the top panel, we see the corresponding shifts for participants who shadowed in the reverse order, starting with the NZ speaker. In general, the bars representing participants in the New Zealand and Australian conditions are highest, which visually supports the finding in Table 3 that there is overall significantly more convergence to the New Zealand model (β = 9 Hz, *p* = 0.038).

**Table 3:** **Summary of best mixed effects model for U.S. Midland Participants for monophthongs**.

	**Estimate**	**Std. error**	***t*-value**	***p*-value**
**MIDLAND SPEAKERS—MONOPHTHONG MODEL (EUCLIDEAN DIFFERENCE IN DISTANCE AT VOWEL midpoint)**				
(Intercept)	6.104	4.213	1.448	0.148
Condition = U.S. Inland North	−2.853	3.486	−0.819	0.413
Condition = Australia	2.660	3.784	0.703	0.412
Condition = New Zealand	8.766	3.903	2.246	0.038^*^
Order = NZ	−2.361	3.889	−0.607	0.544
Word Class = bath	2.857	4.842	0.590	0.555
Word Class = dress	−0.113	4.638	−0.024	0.981
Word Class = lot	−0.121	4.650	−0.026	0.979
Word Class = trap	2.791	4.643	0.601	0.548
Condition = U.S. North: Order= NZ	2.100	3.327	0.631	0.528
Condition = Australia: Order= NZ	−0.024	3.783	−0.006	0.995
Condition = New Zealand: Order= NZ	−4.075	3.473	−1.173	0.606
Condition = U.S. North:: Word class = bath	−5.415	6.420	−0.844	0.399
Condition = Australia: Word class = bath	8.308	6.055	1.372	0.170
Condition = New Zealand: Word class = bath	−11.672	7.034	−1.659	0.191
Condition = U.S. North:: Word class = dress	3.207	6.110	0.525	0.600
Condition = Australia: Word class = dress	1.098	5.797	0.189	0.850
Condition = New Zealand: Word class = dress	−6.110	6.746	−0.906	0.778
Condition = U.S. North: Word class = lot	4.595	6.145	0.748	0.455
Condition = Australia: Word class = lot	3.943	5.781	0.682	0.495
Condition = New Zealand: Word class = lot	−4.245	6.773	−0.627	0.506
Condition = U.S. North: Word class = trap	9.451	6.143	1.538	0.124
Condition = Australia: Word class = trap	−12.929	5.790	−2.233	0.026^*^
Condition = New Zealand: Word class = trap	6.714	6.763	0.993	0.614
Order = NZ: Word class = bath	5.831	3.483	1.674	0.094
Order = NZ: Word class = dress	1.371	3.326	0.412	0.680
Order = NZ: Word class = lot	−0.078	3.342	−0.023	0.981
Order = NZ: Word class = trap	−2.021	3.333	−0.606	0.544
Condition = U.S. North: Order= NZ: Word class = bath	−18.645	6.051	−3.081	0.005^*^
Condition = Australia: Order= NZ: Word class = bath	21.121	6.054	3.489	<0.001^*^
Condition = New Zealand: Order= NZ: Word class = bath	14.583	6.000	2.432	0.002^*^
Condition = U.S. North: Order = NZ: Word class = dress	9.199	5.752	1.599	0.110
Condition = Australia: Order= NZ: Word class = dress	−6.578	5.798	−1.135	0.257
Condition = New Zealand: Order= NZ: Word class = dress	−16.706	5.747	−2.907	0.014^*^
Condition = U.S. North: Order= NZ: Word class = lot	7.575	5.790	1.308	0.191
Condition = Australia: Order= NZ: Word class = lot	−15.759	5.781	2.726-	0.006^*^
Condition = New Zealand: Order= NZ: Word class = lot	−8.740	5.779	−1.512	0.003^*^
Condition = U.S. North: Order= NZ: Word class = trap	3.798	5.787	0.656	0.512
Condition = Australia: Order= NZ: Word class = trap	5.927	5.790	1.024	0.306
Condition = New Zealand: Order= NZ: Word class = trap	−2.239	5.766	−0.388	0.192
**MIDLAND SPEAKERS–PRICE MODEL (EUCLIDEAN DIFFERENCE IN DISTANCE AT VOWEL MIDPOINT)**				
(Intercept)	5.544	7.380	0.751	0.452
**MIDLAND SPEAKERS–NEAR MODEL (DIFFERENCE IN F3 DISTANCE AT VOWEL & /R/ 65% POINT)**				
(Intercept)	69.60	27.38	2.542	0.011^*^
Condition = Australia	83.92	30.61	2.742	0.006^*^
Condition = U.S. Inland North	−78.94	41.78	−1.889	0.059
Condition = New Zealand	30.24	32.50	0.930	0.129

*Dependent variable is the difference in Euclidean distance between model and shadower across baseline and shadowing recordings. Random effects = (1 + Condition |Word) + (1 + Condition |Subject)*.

*Asterisks indicate significance at a 0.05 alpha*.

A significant two-way interaction between condition and class is driven by trap words in the Australian condition, which, are significantly less imitated than other Australian vowel classes (β = −13 Hz, *p* = 0.026). The three-way interaction in the monophthongs model is visible in Figure 6. When shadowing the Australian and New Zealand models last, Americans show no convergence to them on bath, but *do* converge on bath for the Australian (β = 21 Hz, *p* < 0.001) and New Zealand (β = 15 Hz, *p* = 0.002) conditions when they are shadowing these models *first*. For the Inland North condition, the effect is opposite: when the NZ models are first, there is significantly less convergence on bath (β =−19 Hz, *p* = 0.005). In contrast to the effect of order on bath in the Antipodean conditions, when these models are first there is less convergence to dress (β = −17 Hz, *p* = 0.014) and lot (β = −9 Hz, *p* = 0.003) of the NZ model, and less convergence to lot of the Australian model (β = −16 Hz, *p* = 0.006).

**Figure 7:**
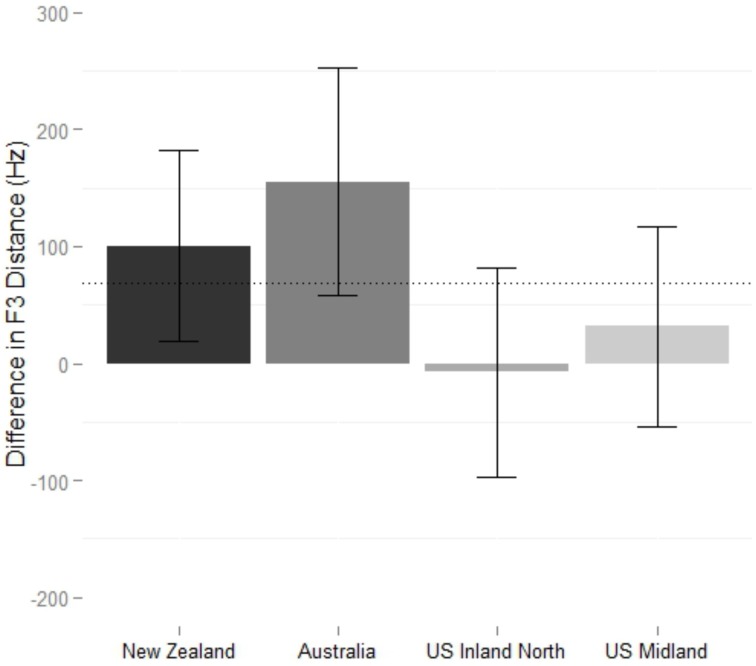
**Difference in F3 distance between Midland shadowers and their model across baseline and shadowed tokens on NEAR**. Error bars mark 95% confidence intervals. Dotted line represents grand mean of model.

None of the factors were significant predictors of price nucleus shift, nor was the grand mean significantly different from zero (Table 3), suggesting no overall convergence. Figure 7 shows the shifts in F3 distance on near, and the final model includes shadowing condition (Table 3). The significant intercept (70 Hz) shows that there was overall convergence on F3 by the Midland participants (*p* = 0.011), and the effect of Condition is driven by the fact that there was exceptional convergence to the Australian model (β = 84 Hz, *p* = 0.006). Unlike the New Zealand near model (the the U.S. monophthong model), including order did not significantly improve the model.

### Experiment one summary

We see convergence in formant measurements in both the American and New Zealand participants, though there is more general convergence by New Zealanders on vowels, and more by the Americans on rhoticity. Both groups of participants show more convergence to the farthest away dialects (supported by the main effect of Condition in both monophthong models and the American rhoticity model). Additionally, there is evidence that general patterns in shifting are mediated by vowel class: New Zealand participants show significantly more convergence on dress when shadowing American participants and significantly less when shadowing Australians. They show more convergence to the Australian than otherwise expected and less to the Northern US speaker for trap. Conversely, a two-way interaction of condition and class for the American participants suggest they show less convergence than expected to Australian trap.

Order additionally impacts convergence, on the bath, dress and lot vowels for the American participants and on rhoticity and trap for the New Zealanders. The rhoticity effect for NZ participants and the bath effect for the Americans might support an interpretation that there is more convergence to the dialects that come first, though the American patterns on dress and lot counter such an interpretation or suggest that it is more complicated.

## Experiment 2: AXB analysis

The acoustic analysis above focuses on the alteration of formant structures, as we are primarily interested in convergence to vocalic quality. However, it is worth establishing whether listeners are able to use such shifts in assessing convergence (Pardo, 2013; Pardo et al., 2013). Additionally, even if listeners are using formant distance to decide whether phonetic convergence occurred, seeing whether some variables are independently heard as eliciting more or less convergence is interesting, suggesting either that listeners are more sensitive to shifts on certain variables over others, or that speakers were accommodating to other acoustic features (for example, pitch or duration) on some variables more than others. To examine possible patterns of convergence not directly tied to the regionally differing formant structures, and to see how sensitive listeners were to the accommodation acoustically established, we constructed a second experiment in which naive American listeners assessed how similar the shadowed tokens were to their models, relative to the shadower's original baseline utterance of the same word.

### Method

The shadowed recordings were average intensity normalized to the same level as the models had been. Every shadowed token was spliced into three-word combined files in one of the two orders baseline-model-shadow or shadow-model-baseline. These combined recordings were uploaded to Mechanical Turk, a crowdsourcing marketplace where “requestors” post simple tasks requiring human intelligence and “workers” perform the tasks (for a discussion of the use of Mechanical Turk in linguistics research, see Callison-Burch and Dredze, 2010; Sprouse, 2011). A single AXB judgment was presented as an individual task, for U.S.$0.03 per judgment, which took typically 5 s or less. Only judges from the US were used. Due to Amazon's payment structure, which is most straightforward for workers in the US and India, collection from New Zealand judges was not feasible. No judges from other countries were used. Participants heard these files along with a screen asking them to select via binary forced choice whether the first or the third item was most similar to the middle item.

Every shadowed token was judged once, half with the shadowed token preceding the model and half with it following. Judges were allowed to perform as many of the tasks as they liked, resulting in data from 86 judges who judged from 1 to 681 ordered trios each (mean 115; median 48). Due to technical complications, a subset (26%) of judges heard only tokens from US Midland shadowers while a much smaller number (4%) heard only tokens from New Zealand shadowers. In the models below, we only present data from the judges who heard tokens from both New Zealand and American shadowers.

The methodological choice to have each token judged once is somewhat unusual in accommodation research. For example, Pardo (2013) states the field's standard as being 5–30 AXB judgments per token. The limitation of this choice is that conclusions often rest on smaller samples of *shadowers*, for example, 12 total (3 per gender^*^role cell) in Pardo (2006), even when the focus of the investigation is on the shadowers' behavior. We have here prioritized number of shadowers, including all 37 from Experiment 1, and we make intra-speaker comparisons across conditions. So while this leaves us with a small per-token judgment count, our more statistically crucial number of judgments per cell is around 170–200 for each class^*^condition^*^place combination.

Because we wanted to include the acoustic measures used in our acoustic analysis, we built separate mixed effects logistic regression models for the monophthongs, price, and near. Each model was fit to the responses of the Mechanical Turk judges (did they choose the shadowed over the baseline recording) and included random effects for shadower and judge (with random slopes for significant fixed effects). An additional random intercept of lexical item was tested and found not to improve the models. Fixed effects tested, using sum contrasts, were word class (base group kit), shadowing condition (base group U.S.), shadower national origin (base group U.S.), shadowing block order, and AXB order. The acoustic measures used in the first part (Euclidean distance in F1-F2 space taken at the midpoint for the monophthongs, taken at the vowel nucleus for price, and the F3 difference taken at 65% of the rhyme for near) were also included as numerical variables and were uncentered because of their meaningful zero. Two three-way interactions were also tested, between shadow block order, word class and, on the one hand, speaker national origin and, on the other, shadowing condition. Items were included based on model comparison, retaining those which significantly (α = 0.05) improved the model as a whole.

### Results

Table 4 shows the best fit model for the monophthongs. Two main effects were retained as significant in the full model. First, a strong bias on the part of the judges in favor of the third token over the first token in the AXB task, leading to a significant decrease in responses supporting convergence when the first token was the shadowed one (*p* = 0.007). Second, the larger the shift in Euclidean F1-F2 toward the model, the more likely listeners were to choose the shadowed token (*p* = 0.015). This confirms that the measurements in the acoustic task were capturing shifts that listeners were sensitive to. The fact that the intercept–representing the grand mean when the Euclidean distance equals zero–is significant (*p* = 0.013), suggests that there are other things in the signal, beyond Euclidean distance, that listeners are using to choose the shadowed token more often than chance (for example, duration, voice quality, etc.). However, the small overall mean (52.67%) suggests that either the accommodation or the listeners' abilities to detect it was slight. This is consistent with other studies, where proportions of shadowed tokens chosen rarely exceed 60%.

**Table 4:** **Summary of best mixed effects logistic regression model for AXB task**.

	**Estimate**	**Std. error**	***z*-value**	***P*-value**
**NZ MONOPHTHONG MODEL (DIFFERENCE IN EUCLIDEAN DISTANCE**				
**AT VOWEL MIDPOINT)**				
(Intercept)	0.10687	0.04287	2.493	0.013^*^
Difference in F1-F2 Distance	0.08422	0.03460	2.434	0.015^*^
AXB order = First	−0.25987	0.09591	−2.709	0.007^*^
**NZ PRICE MODEL (DIFFERENCE IN EUCLIDEAN DISTANCE AT VOWEL 20%)**				
(Intercept)	0.05581	0.07679	0.727	0.467
Difference in F1-F2 Distance	−0.10800	0.06734	−1.604	0.109
AXB order = First	−0.18947	0.13538	−1.400	0.162
**NZ NEAR MODEL (DIFFERENCE IN F3 DISTANCE AT RHYME 65%)**				
(Intercept)	0.01640	0.08003	0.205	0.838
Difference in F3 Distance	0.09238	0.06513	1.418	0.156
AXB order = First	−0.12402	0.12987	−0.955	0.340

*Dependent variable is whether the shadowed or baseline token was selected (0 = baseline, 1 = shadowed token). Random effects = (1+AXB Order+ Difference |Speaker) + (0+AXB Order + Difference |Judge)*.

*Asterisks indicate significance at a 0.05 alpha*.

In models for price and near, the acoustic measures used in Experiment 1 significantly improve the models, but do not reach significance on their own, suggesting that listeners may not have been using these cues or that the effect was too small to be detected given our sample (or, especially in the case of price, that there were no changes in the cue to be heard). AXB order also improves the models, but is not significant on its own. Neither intercepts were significant, suggesting that participants did not hear convergence in general for either variable (or again, that the sample was too small).

### Summary of AXB

Participants in the AXB task heard convergence in the monophthongs, but the effect size was small, and the clearest factor influencing AXB decisions was the AXB order. The acoustic measures improved all models and were significant within the model for the monophthongs, suggesting that listeners used changes in F1-F2 to make their choices. There was no evidence that the condition, shadower origin, or block presentation in the shadowing task affected how much listeners heard convergence.

## General discussion

In this study we compared how shadowers from two dialect regions shadowed speakers from four dialect regions, across seven word classes. The results presented here offer additional evidence that speakers can and do converge toward speakers of other dialects, even in a socially impoverished task like single-word shadowing, where the shadowers have been instructed not to alter their speech. Specifically, we see shifts in formant values that we interpret as changes in vocalic quality and rhoticity, and the AXB task confirms that the vocalic shifts are changes that listeners are sensitive to. However, whether we observe a shift, and how big it is, depends on the dialect of the speaker, the dialect they are shadowing, and the variable in question. Additionally, some of these results are further complicated by an order effect. The general patterns in the data support arguments that phonetic distance, phonetic repertoire, and saliency matter, but also suggest that other, primarily social, factors are influencing speakers at a variable-specific level.

There are two types of evidence that suggest that the size of the original distance between two speakers matters in observing convergence, with larger differences leading to larger shifts toward a model speaker. The first is that we generally see more convergence by speakers to the dialects most different than their own. The second is that the variable on which New Zealanders shift the most–dress—is one of the variables that differs most between the New Zealand participants and the American models.

This first point contrasts with findings by Kim et al. (2011), whose participants showed convergence within, but not across dialects. In our study, we find accommodation to the most distant dialects and observe maintenance in speakers shadowing their own or similar dialects. In this way, our findings support Babel (2012), who argues that “greater phonetic distance, which is a function of dialect background, seems to allow for more imitation” (p. 187). There are a number of methodological differences between Kim et al.'s study and our own, including quantitatively and qualitatively different dialects boundaries involved (L1-L2 speakers and Americans-Americans vs. Americans-Antipodeans), different tasks to elicit accommodation (interactive diapix task vs. shadowing), different primary analysis (acoustic vs. AXB), and different AXB instructions^7^. The results strongly suggest that more work comparing accommodation across different types of dialect boundaries, under different circumstances, is necessary to elucidate the reasons for the different outcomes of these tasks.

New Zealanders exhibit exceptional shifting on dress toward American speakers, similar to the large shifts seen in New Zealanders in Babel (2010), though, unlike Babel's, our New Zealanders do not exhibit this shift when shadowing Australians, and instead actually show an exceptional lack of shift to Australian (and New Zealand) dress. This may be due to the relatively raised and fronted dress of the New Zealand model, and the fronted DRESS productions of our Australian model, compared to the New Zealand baseline (Figure 4). The difference then between the US and Antipodean models (and our results and Babel (2010)) suggests that their flexibility on this variable is direction specific (c.f. Nielsen, 2011), either due to the structure of the change in progress itself, or the social associations of extremely raised and fronted dress tokens within New Zealand (Drager, 2005).

While dress was one of the variables in which we saw the biggest differences between the models and the participants, there were other variables, like kit, bath, and trap, where there were considerably large distances between models and participants (Table 1). Additionally, while New Zealanders exhibited exceptional shifting on dress to American models, the Americans did not reciprocate the shift. Why are New Zealanders so shiftable (by lowering) on dress specifically?

While Trudgill (1981) has argued that we will see the largest shifts where there are larger differences, he also said they will be the most salient differences. Babel's (2010) results, and the results here suggest otherwise, given that dress is considered to be below the level of consciousness to New Zealanders (Maclagan et al., 1999), in contrast to the widely known Australian-New Zealand kit vowel difference, which has reached the level of a sociolinguistic stereotype, in Labov's (1972) terminology (Bayard, 2000; Hay et al., 2006). Indeed, while the acoustic models support the idea that New Zealanders shift more on dress than Midlanders, only 2/20 New Zealanders mentioned the dress vowel as a noticeable difference when shadowing the Americans, compared to a majority of the Midland participants who noted this variable. And New Zealanders who correctly identified the Australian model as Australian often cited the kit vowel as the reason for their decision. This suggests that it is critically the combination of distance and lack of awareness which promotes convergence to recently heard tokens [as argued by Babel (2010)].

This pattern contrasts interestingly with Drager et al. (2010) evidence of shifts on kit but not dress or trap by New Zealanders in an Australian dialect priming task. These speakers were not exposed to Australian speech but rather to the conceptual category of Australia. This may suggest that direct linguistic input affects speech differently than priming a dialect. It would not be surprising if speaker awareness of linguistic differences were required for the latter, given that speakers must themselves make the link between the conceptual representation of place (i.e., “Australia”) and the linguistic details (i.e., raised productions of kit).

Another explanation for why dress is so special for New Zealanders is that, as argued by Babel (2012), a person's existing phonetic space may impact where they are likely to shift. In this sense, speakers may shift on vowels if the shift is consistent with ways they already have experienced saying that vowel. In Babel's example, she argued low vowels observed more convergence because they have inherently larger production spaces, although our study does not replicate this pattern of low vowels specifically. However, the raising of dress in New Zealand English is a change in progress in New Zealand (Maclagan and Hay, 2004), and when vowels are changing within the population, individuals also show greater variability (Trudgill et al., 1998; Gordon and Maclagan, 2001). Thus New Zealanders may have more personal flexibility in this vowel class, in a way that American speakers do not, leading them to accommodate more easily on it. Phonetic repertoires can also explain why Americans show greater convergence in rhoticity than the New Zealanders do, because rhotic systems inherently have more variability than non-rhotic systems due to reduction in unstressed positions (Scobbie, 2006; Piercy and Britain, 2012).

The order effects we observe complicate these other explanations. For New Zealanders, we find that they converge to trap in general and American rhoticity, when the shadow the American models first. For American participants, we see more convergence to the Antipodean models on dress and lot when these models come last, to bath when these models come first, but we see less convergence to the Inland North on bath when the Antipodeans are first. The phonetic distance of a speaker's baseline productions to the models' and their baseline phonetic repertoires do not differ across the order of presentation, so these explanations cannot explain what we see here.

We did not set out to test for order; we manipulated it as a control, and so further research will be necessary to determine whether order of presentation is a general factor influencing cross-dialect accommodation. It is worth reflecting on what such an effect might mean, should it prove to be real. Order of dialect presentation may impact the context in which each accent is heard, making some variables socially significant/contrastive in a way that might not have been in a different order. For example, New Zealanders listening to rhotic Americans may be more aware of rhoticity as *Not New Zealand English* if they have just shadowed New Zealanders, compared to if they started with Americans. It is also possible that the order effect is in fact a fatigue effect, where participants may simply have been more tired by the later blocks, leading to greater accommodation as the experiment progressed (though this alone does not account for all the order effects we observe). It has been argued that imitation is a default mechanism both in language and in social interaction more generally, across species, and that it takes brain function to inhibit accommodation (Dijksterhuis and Bargh, 2001), which may be harder as participants become fatigued by the task. Note that other findings that participants converge more toward the end of the task have often been explained in terms of people getting exposure to the speaker (i.e., Babel, 2009), but that cannot be the case in our task where the models changed as the experiment progressed.

Whatever the explanation for the order effect, we think it is non-trivial that for American speakers, the order effect generally shows more convergence toward Antipodeans later in the task except on bath, where there is more convergence to the Antipodeans if they are first. bath was an interesting inclusion in this study. The difference between the Antipodean and American dialects here is phonological, not phonetic (though so is rhoticity), and Trudgill has argued that accommodation processes are phonetic and not phonological Trudgill (1986). But in addition, bath carries heavy social loading, especially in America: in the U.S. and Canada, the production of an [a] as opposed to [æ] is commonly accepted as a more “correct,” “authentic,” and politically liberal production of nativized foreign words including an orthographic <a> (Boberg, 1999; Hall-Lew et al., 2010). Indeed, we informally observed more channel cues—delays, giggles and false starts—when American participants were saying bath words. Abrego-Collier et al. (2011) and Babel (2010, 2012) have shown that a shadower's attitude toward their interlocutor and his/her ethnolinguistic group may mediate the degree of convergence even in a socially impoverished task; here the social saliency of this variable then may be responsible for its distinctive patterning.

As shown by comparing Figures 3–6, New Zealand participants converge more to Americans than U.S. Midland speakers converge to the Antipodean speakers on vocalic measures, independent of order. New Zealanders have a large amount of exposure to American accents through popular media^8^, and while they could not tell the U.S. Inland North and U.S. Midland speaker apart very much, they always knew the speakers were North American, compared to a 6% correct identification of the New Zealand speaker and 44% correct identification of the Australian by U.S. Midland participants. This exposure, which we could consider a passive phonetic repertoire, appears to have made New Zealand participants either more able, or more willing, to converge to American speakers (cf. Nye and Fowler, 2003).

## Conclusion

This study compares cross-dialectal accommodation by two groups of speakers to four different dialect regions. Specifically, we measured vocalic accommodation in terms of F1-F2 of six vowels, and F3 in rhotic environments. We observed convergence, but not consistently across vowel classes, speaker-dialect, participant-dialect, or task order. Instead, while the complicated results support previous claims that the phonetic distance of the vowel from the shadower's own productions, the shadower's phonetic repertoire matter, and saliency matter, they also show that other factors must also be at play, and that considering the social associations and context of particular variables may play a role even in these relatively reduced social circumstances. A closer integration of work on accommodation and work on socio-indexical meaning of linguistic detail may be promising as a route for future work.

### Conflict of interest statement

The authors declare that the research was conducted in the absence of any commercial or financial relationships that could be construed as a potential conflict of interest.

## Footnotes

^1^It has also been argued that such an automatic process might be inhibited by processing and attentional demands in producing and perceiving non-native speech (Costa et al., 2008; Kim et al., 2011).

^2^We use Wells' (1982) lexical sets to refer to vowel classes; each word in small caps refers to a class of words which share the same vowel in most, though often not all, varieties of English.

^3^One bath class word, *chant*, was removed from analysis due to complicating factor of pre-nasal raising in American English.

^4^And, as a reviewer points out, they may have engaged in more subvocal or internal rehearsal to help judge the distinctiveness of the model's variety.

^5^And it was necessary: in a pilot version of the study without this instruction, a participant started explicitly imitating the presented dialects.

^6^For effect coding, the base group should be the least interesting level of a variable. In this case, all levels of vowel class were theoretically interesting. However, a previous model using treatment coding suggested that we generally see the least convergence and interactions for KIT, for both American and New Zealand participants (see Figures 3, 6).

^7^While ultimately both sets of participants had been asked to choose the token (A/B) that was most similar to X, Kim et al. framed the question in the context of explicit imitation, telling participants to “imagine that the second talker (in samples A and B) was attempting to impersonate the model talker (in sample X)” (p133).

^8^In 1999, American television content was 90% American compared to New Zealand television content being 24% kiwi; in 2009 in New Zealand it was 39% kiwi (NZ On Air, 1999, 2010).

## Appendix

APPENDIX A: List of words in the shadowing task, organized by word class. CELEX log wordform frequency in parentheses (Baayen et al., 1995).

trap (mean freq = 0.76368)

bad (2.3201), bash (0), clap (0), crab (.6021), flag (.9542), flash (1.5051), sash (.301), mat(1.4772), slap(.4771), smack(0)

bath (mean freq = 1.54925)

chant (.301)^*^, clasp (0), craft(.8451), fast(3.6169), last (4.8942), mast(.4771), raft(.4771), shaft(1), staff(2.0682), task(1.8129)

dress (mean freq = 0.95332)

bed (2.3874), deaf(1.2552), deck(1.2788), fresh(1.8513), guest(1.3802), jet(1.0792), mesh (0.6021), sled (0), sledge (0), wedge (0.699)

price (mean freq = 1.29577)

bright (2.1761), dive (0.301), glide (0), hide (1.3222), knife (1.5441), size (2.0492), slight(1.5441), snide (0), wide (3.1179), vice (0.9031)

near (mean freq = 1.93293)

beer (1.6628), clear (3.3444), fear (3.0607), gear (1.3617), near (5.6375), peer (0.6021), smear (0.4771), sneer (0), tier(0.4771), year (2.7059)

lot (mean freq = 0.73211)

boss (1.3424), chop (0.4771), dock (1), flock (0.8451), froth (0.301), loft (0.4771), mop(0.301), notch (0.301), scotch (1.4313), squash (.8451)

kit (mean freq = 0.79431)

bib (0.301), dig (1.0792), drip (0.301), gift (1.4914), hiss (0.301), mist (1.1139), ridge (1.0792), rip (0), sniff (0), thick (2.2764)

^*^*chant* was removed from analysis because of complications due to pre-nasal /æ/-raising in American English.
